# Genes involved in the limited spread of SARS-CoV-2 in the lower respiratory airways of hamsters may be associated with adaptive evolution

**DOI:** 10.1128/jvi.01784-23

**Published:** 2024-04-16

**Authors:** Kosuke Takada, Yasuko Orba, Yurie Kida, Jiaqi Wu, Chikako Ono, Yoshiharu Matsuura, So Nakagawa, Hirofumi Sawa, Tokiko Watanabe

**Affiliations:** 1 Department of Molecular Virology, Research Institute for Microbial Diseases, Osaka University, Suita, Osaka, Japan; 2 Division of Molecular Pathobiology, Research Center for Zoonosis Control, Hokkaido University, Sapporo, Hokkaido, Japan; 3 International Collaboration Unit, Research Center for Zoonosis Control, Hokkaido University, Sapporo, Hokkaido, Japan; 4 One Health Research Center, Hokkaido University, Sapporo, Hokkaido, Japan; 5 Department of Molecular Life Science, Tokai University School of Medicine, Isehara, Kanagawa, Japan; 6 Laboratory of Virus Control, Research Institute for Microbial Diseases, Osaka University, Suita, Osaka, Japan; 7 Center for Infectious Disease Education and Research, Osaka University, Suita, Osaka, Japan; 8 Bioinformation and DDBJ Center, National Institute of Genetics, Mishima, Shizuoka, Japan; 9 Institute for Vaccine Research and Development, Hokkaido University, Sapporo, Hokkaido, Japan; 10 Global Virus Network, Baltimore, Maryland, USA; 11 Center for Advanced Modalities and DDS, Osaka University, Suita, Osaka, Japan; Emory University School of Medicine, Atlanta, Georgia, USA

**Keywords:** evolution, coronavirus, animal models

## Abstract

Novel respiratory viruses can cause a pandemic and then evolve to coexist with humans. The Omicron strain of severe acute respiratory syndrome coronavirus 2 has spread worldwide since its emergence in late 2021, and its sub-lineages are now established in human society. Compared to previous strains, Omicron is markedly less invasive in the lungs and causes less severe disease. One reason for this is that humans are acquiring immunity through previous infection and vaccination, but the nature of the virus itself is also changing. Using our newly established low-volume inoculation system, which reflects natural human infection, we show that the Omicron strain spreads less efficiently into the lungs of hamsters compared with an earlier Wuhan strain. Furthermore, by characterizing chimeric viruses with the Omicron gene in the Wuhan strain genetic background and *vice versa*, we found that viral genes downstream of ORF3a, but not the S gene, were responsible for the limited spread of the Omicron strain in the lower airways of the virus-infected hamsters. Moreover, molecular evolutionary analysis of SARS-CoV-2 revealed a positive selection of genes downstream of ORF3a (M and E genes). Our findings provide insight into the adaptive evolution of the virus in humans during the pandemic convergence phase.

**IMPORTANCE:**

The severe acute respiratory syndrome coronavirus 2 (SARS-CoV-2) Omicron variant has spread worldwide since its emergence in late 2021, and its sub-lineages are established in human society. Compared to previous strains, the Omicron strain is less invasive in the lower respiratory tract, including the lungs, and causes less severe disease; however, the mechanistic basis for its restricted replication in the lower airways is poorly understood. In this study, using a newly established low-volume inoculation system that reflects natural human infection, we demonstrated that the Omicron strain spreads less efficiently into the lungs of hamsters compared with an earlier Wuhan strain and found that viral genes downstream of ORF3a are responsible for replication restriction in the lower respiratory tract of Omicron-infected hamsters. Furthermore, we detected a positive selection of genes downstream of ORF3a (especially the M and E genes) in SARS-CoV-2, suggesting that these genes may undergo adaptive changes in humans.

## INTRODUCTION

Pandemics cause such enormous damage that they disrupt the order of human society, but once the pandemic is brought under control, social order is restored. Factors such as the establishment of prevention and treatment methods for infectious diseases and attaining population or herd immunity play a major role in the convergence of pandemics. In addition, pandemics sometimes converge when viruses follow an adaptive evolutionary path of symbiosis with a new human host. One specific example is an influenza pandemic (1). An animal-derived influenza virus (usually a reassortant virus) spreads to humans, infects a human population that has no immunity to the new virus, causes a pandemic, and then becomes a seasonal influenza virus that becomes established in the human world (2–4). After that, it becomes an epidemic once a year during the winter season. At that time, the virus itself may undergo adaptive evolution with weakening. The 1918 influenza pandemic, which killed an estimated 40 million people worldwide, caused many deaths from pneumonia at the time of the pandemic (1); however, its descendant, the seasonal H1N1 influenza virus, causes mainly upper respiratory tract infections and seldom causes pneumonia (5). Yet, there are many unknowns regarding how viruses evolve from pandemic viruses to seasonal ones, particularly during the convergence process.

With respect to coronaviruses, four human coronaviruses are currently known as seasonal viruses (6, 7). Gene sequence analysis suggests that these viruses were originally animal coronaviruses that became established in human society as seasonal coronaviruses after zoonotic pathogens spread in humans and probably caused a pandemic (7, 8), but the actual course they followed is unknown. In the 21st century, severe acute respiratory syndrome (SARS) coronavirus, Middle East respiratory syndrome (MERS) coronavirus, and severe acute respiratory syndrome coronavirus 2 (SARS-CoV-2) emerged as zoonotic coronaviruses infecting humans, with SARS coronavirus causing an outbreak that was successfully contained, and MERS coronavirus transiently transmitting from camels to humans (7, 9). However, efficient human-to-human transmission of these two viruses has not been reported; only SARS-CoV-2 has caused a pandemic.

Since its emergence in China in 2019, SARS-CoV-2 has genetically evolved during repeated human infections, producing numerous genetic variants (10). The Omicron strain (BA.1 lineage), which emerged in late 2021, spread worldwide at a tremendous rate, and now sub-lineages are emerging and becoming established in human society (11). The most common clinical symptoms seen in patients infected with Omicron strains are respiratory symptoms of the upper respiratory tract, including sore throat, sneezing, nasal congestion, and runny nose (12, 13). It has also been reported that the Omicron strain is markedly less invasive to the lungs and other lower respiratory tracts and causes less severe disease in patients compared with previous strains (12). One reason for this is that humans are acquiring collective immunity through previous infection and vaccination, but it is also possible that the virus itself is changing. Previous studies have shown that the Omicron strain has lower growth efficiency than the Wuhan and Delta strains in cultured cells derived from human lungs (14). Furthermore, Hui et al. (15) reported that Omicron strains replicate more efficiently in human bronchi than Wuhan, Alpha, Beta, and Delta strains, but less efficiently in human lungs. In addition, several studies have shown that the Omicron subtype replicates less efficiently in mouse and hamster lungs than Wuhan, Beta, and Delta strains but replicates at the same level in the nasal conduits (14, 16, 17). The Omicron strain has at least 30 substitutions and deletions in its S protein, but several unique amino acid substitutions have also been detected in its other viral proteins (18). However, the relationship between the Omicron-specific amino acid substitutions and viral properties has not been fully investigated, and the detailed mechanisms remain unclear.

The initial target of SARS-CoV-2 infection is the upper respiratory tract, particularly nasal mucosal epithelial cells (19–21). Therefore, the virus is thought to spread first in the upper respiratory tract and then to the lower respiratory tract, including the lungs (22). Although hamsters are an animal model that reflects human infection, the conventional infection method involves inoculating a large amount of virus (30–100 µL) through the nose, which causes the virus solution to reach the lower respiratory tract during inoculation (14, 16, 17, 23), as previously reported for influenza viruses in a ferret model (24). Therefore, to establish an animal model that more closely reflects natural infection in humans, we developed an infection system using low-volume nasal inoculation of hamsters. Using this system, we performed a comparative analysis of the replicative ability of Omicron and Wuhan strains in the respiratory tract and showed that the Omicron strain replicated less efficiently in the lower respiratory tract. Furthermore, by generating a series of chimeric viruses of the Omicron and Wuhan strains and examining their replicative ability in the hamster respiratory tract, we found that genes downstream of ORF3a, but not S, are involved in the limited spread of SARS-CoV-2 in the lower airway of hamsters. Evolutionary analysis of the gene region after ORF3a showed that positive natural selection is at work in the Omicron-specific amino acid substitutions at E and M. Our findings provide insights into the adaptive evolution of viruses in humans during the transitional phase of a pandemic virus to a seasonal virus at pandemic convergence.

## RESULTS

### Establishment of a low-volume SARS-CoV-2 infection system, which mimics natural human infection, in the hamster model

**Fig 1:**
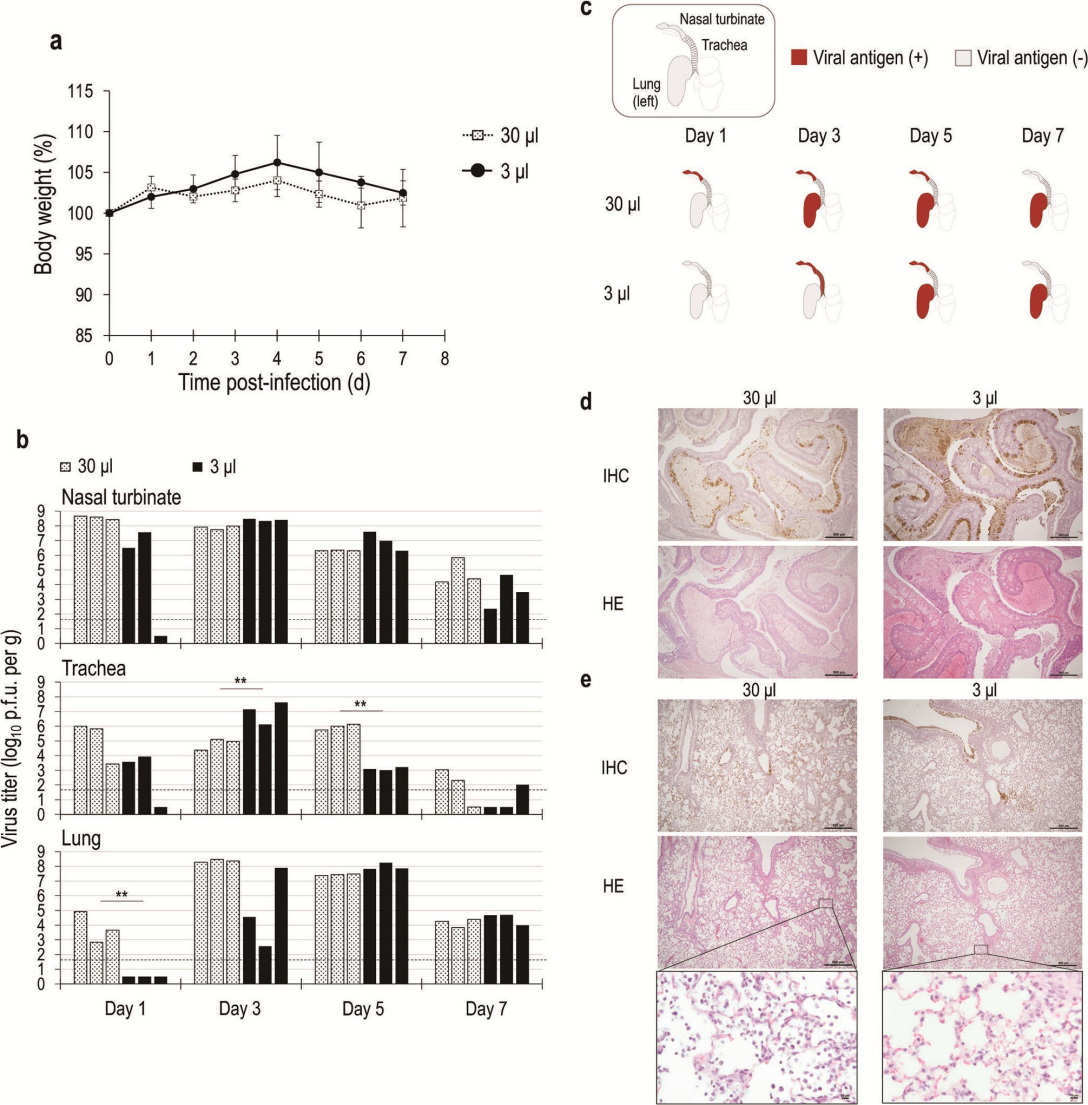
Comparison of the low- and high-virus-volume hamster infection systems using Wuhan strains. Wild-type Syrian hamsters were intranasally inoculated with 10^3^ PFU in 30 µL (high virus volume) or 3 µL (low virus volume) of recombinant Wuhan strain. (a) Body weights of virus-infected (*n* = 12) animals were monitored daily for up to 7 days. Note that three animals were euthanized in each group at 1-, 3-, and 5-days post-infection. Data are presented as the mean percentages of the starting weight (±SD). (b) Virus titers in the organs of hamsters infected with each virus. Three hamsters per group were euthanized at 1-, 3-, 5-, and 7-days post-infection for virus titration. Virus titers in the nasal turbinate, trachea, and lung were determined by means of plaque assays in VeroE6/TMPRSS2 cells. Vertical bars show the virus titer, and the same row indicates the same individual hamsters. The detection limit was set at 0.5 (log_10_ PFU/g). Mean values were compared by an analysis of variance, followed by Dunnett’s test (*P < 0.05 and **P < 0.01). (c) Immunohistochemical (IHC) detection of the SARS-CoV-2 nucleocapsid (N) antigen in the nasal turbinate, trachea, and lung of two individuals in each group. Viral antigen-positive regions are indicated in red. (d and e) Histopathological examination of the lungs, trachea, and nasal turbinate of an infected hamster at 5 days post-infection. (d and e) Representative results of IHC analysis for SARS-CoV-2 (N) antigen or hematoxylin and eosin (HE) staining images of infected nasal turbinate (**d**) and (**e**) lungs of hamsters infected with the Wuhan strain at 5 days post-infection using the high-volume (left panel) or low-volume infection system (right panel). Scale bars, 0.5 mm.

Because the conventional hamster animal model uses nasal inoculation of a high volume of virus-containing solution and thus fails to mimic natural human infection, we first compared low-volume and high-volume conditions. Hamsters (three animals per group) were nasally inoculated with either a high volume (30 µL) or low volume (3 µL) containing 10^3^ plaque-forming unit (PFU) of the Wuhan strain (hCoV-19/Japan/TY-WK-521/2020) (note that the inoculum volumes used in this study were determined on the basis of the results of a comparison of different infection volumes in a hamster model; see supplemental material). Body weight changes were observed for up to 7 days post-infection (dpi). On 1, 3, 5, and 7 dpi, hamster nasal turbinate, trachea, and lungs were collected, and virus titers were determined. No significant body weight changes were observed in hamsters in either the 30- or 3-µL infection system (Fig. 1a). Virus was detected in the nasal turbinate and trachea at 1 dpi in all hamsters, except for one hamster infected with 3 µL of virus fluid. Virus was detected in the lungs at 1 dpi in all three hamsters in the 30-μL-infected group, but not in any of the 3-μL-infected hamsters (Fig. 1b). The 30-µL infection group had a peak viral titer of 10^8.4^ PFU/g in the lungs at 3 dpi. In contrast, the 3-μL-infected group had a peak viral titer of 10^8.0^ PFU/g in the lungs at 5 dpi. Furthermore, to determine the distribution of the inoculum in the hamsters, we inoculated hamsters intranasally with tissue-marking dye in a volume of 30 or 3 µL (see supplemental material). Our results showed that inoculation of 30 µL of liquid is sufficient to reach the esophagus and that microscopic droplets could reach the lower respiratory tract. These results suggest that this low-virus-volume infection system in hamsters better reflects natural infections. In the 3-µL infection system, viruses that replicated in the nasal area seemed to be expanded into the lungs of the hamsters.

Next, we performed immunohistochemistry (IHC) analysis of the airways and lungs of the infected hamsters (Fig. 1c through e; Table S1). IHC analysis of the viral nucleocapsid (N) protein was conducted using the upper and lower respiratory organs from randomly selected samples (Table S1). On 1 dpi, viral antigens were detected in the nasal turbinate with the 30-µL infection system, but not with the low-volume infection system. Viral antigens were detected in nasal mucosal epithelium and alveolar epithelial cells at 3 dpi with the 30-µL infection system. In contrast, with the 3-µL infection system, viral antigens were detected in the nasal mucosal epithelium and trachea, but not in the alveolar epithelial cells. Viral antigen was detected in the alveolar epithelium at 5 and 7 dpi in animals inoculated with the Wuhan strain in both the 30- and 3-µL infection system. Pathological features were compared in detail on 5 dpi when the virus was detected in the lungs of hamsters in either the 30- or 3-µL infection system (Fig. 1d and e). In the 30-µL infection system, viral antigens were diffusely detected in a wide area of the lung, whereas in the 3-µL infection system, viral antigens were mainly detected in the bronchial epithelium and alveolar area surrounding the bronchus of the lung (Fig. 1e). Furthermore, hematoxylin and eosin (HE) staining showed that inflammatory cells accumulated over a wide area of the lung in the 30-µL infection system. In contrast, inflammatory cells were mainly observed in the alveolar area surrounding the bronchus of the lung in the 3-µL infection system (Fig. 1e). During natural infection in humans, the first target cells of SARS-CoV-2 are the ciliated airway cells of the nasopharynx and trachea or the sustentacular (non-neuronal) cells of the nasal olfactory mucosa (19–21). If the virus is not eliminated by the host response, replicating viruses can spread to the lung by inhalation of viruses from the upper respiratory tract or by gradual dissemination in the tracheobronchial tubes (22). These results suggest that this low-virus-volume hamster infection system better reflects natural infections.

### Limited replication of Omicron strains in the lower respiratory tract

**Fig 2:**
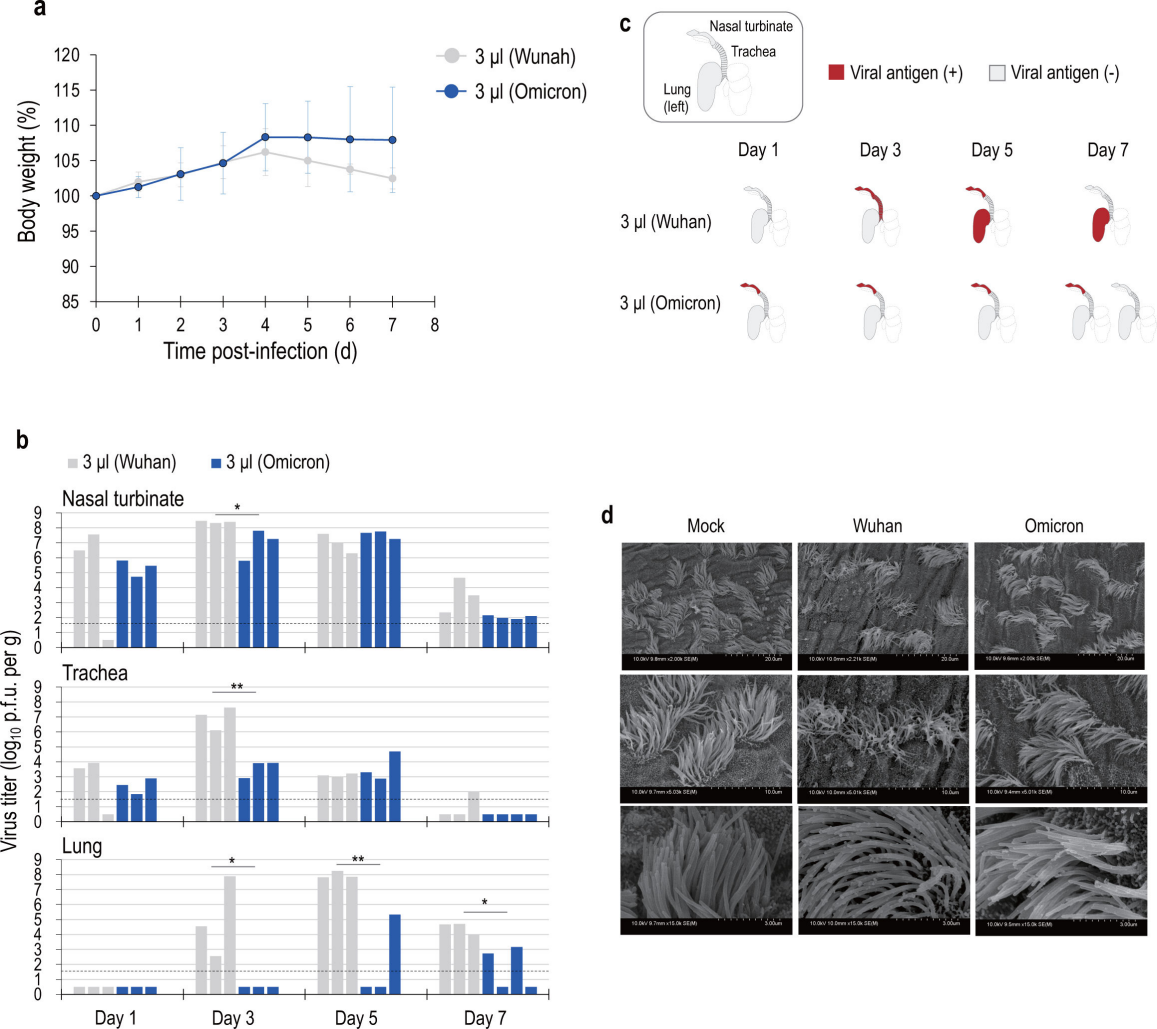
Low-virus-volume hamster infection system using Omicron strains. Wild-type Syrian hamsters were intranasally inoculated with 10^3^ PFU in 3 µL (low virus volume) of recombinant Omicron strain. Note, the Wuhan results are shown next to the Omicron results for comparison; these Wuhan data are the same as those shown in Fig. 1. (a) Body weights of virus-infected (*n* = 13) animals were monitored daily for up to 7 days. Note that three animals were euthanized in each group at 1-, 3-, and 5-days post-infection. Data are presented as the mean percentages of the starting weight (±SD). (b) Virus titers in the organs of hamsters infected with each virus. Three hamsters per group were euthanized at 1-, 3-, 5-, and 7-days post-infection for virus titration. Virus titers in the nasal turbinate, trachea, and lungs were determined by means of plaque assays in VeroE6/TMPRSS2 cells. Vertical bars show the virus titer, and the same row indicates the same individual hamsters. The detection limit was set at 0.5 (log_10_ PFU/g). Mean values were compared by an analysis of variance, followed by Dunnett’s test (*P < 0.05 and **P < 0.01). (c) IHC analysis for SARS-CoV-2 (N) antigen in the nasal turbinate, trachea, and lungs of two individuals in each group. Viral antigen-positive regions are indicated in red. (d) SEM images of tracheal epithelia from mock-, Wuhan strain-, and Omicron strain-infected hamsters at 3 days post-infection.

The Omicron strain has at least 30 substitutions in the S protein, suggesting that its properties may be significantly changed compared with those of earlier strains. In particular, the Omicron strains show reduced replication efficiency in human lungs compared with earlier strains (14, 16, 17). To investigate whether this limited viral replication in the lower respiratory tract can be reflected in the hamster model with our low-virus-volume infection system, we investigated the replication of the Omicron strain (based on hCoV-19/Japan/TY38-873/2021) using our low-volume infection system. No marked body weight loss was observed in hamsters in the 3-µL infection system (Fig. 2a). Virus was detected in the nasal turbinate and trachea at 1-day pi in all hamsters in the 3-µL infection system, but not in their lungs. The viral titers in the lungs of all three hamsters on 3 dpi were below the detection limit in the 3-µL infection system (Fig. 2b). Furthermore, in the lungs, the titers were remarkably lower than those of the Wuhan strain. IHC analysis of the nasal turbinate, trachea, and lungs of the Omicron-infected hamsters in the 3-µL infection system failed to detect viral N protein in the alveolar epithelium at any timepoint tested (Fig. 2c; Table S1). These results indicate that the Omicron strain spreads to the lungs less efficiently.

### Hamster tracheal cilia damage caused by the Omicron strain is milder than that caused by the Wuhan strain

The airway epithelium plays an important role in defense against infection (25). The epithelium is composed of goblet cells, which secrete protective mucus, and ciliated cells, which occupy more than half of the epithelium. Ciliated cells move the mucus layer into the laryngopharynx by rhythmically beating, ultimately allowing it to be swallowed (26). This mechanism of mucus clearance prevents the accumulation of particles and mucus in the lungs.

Previous studies have reported that loss of motile cilia in hamsters infected with SARS-CoV-2 (Wuhan strain) may facilitate the spread of SARS-CoV-2 into the lung parenchyma (27). We, therefore, examined whether the amount of ciliated epithelial damage in the trachea of hamsters differed depending on whether they were infected with the Wuhan or Omicron strain. Hamsters were infected with 3 µL (10^3^ PFU) of Wuhan or Omicron strain via the intranasal route and euthanized at 3 dpi. SEM imaging showed that cilia occupied almost half of the epithelial surface in the trachea of mock hamsters (Fig. 2d). In contrast, there was marked cilia loss in the trachea of hamsters infected with the Wuhan strain, but not the Omicron strain (Fig. 2d). These results indicate that the Omicron strain is less susceptible to ciliated epithelial damage compared to the Wuhan strain.

### The spike protein of the Omicron strain is not responsible for the limited replication of SARS-CoV-2 in the lower respiratory airways of hamsters

**Fig 3:**
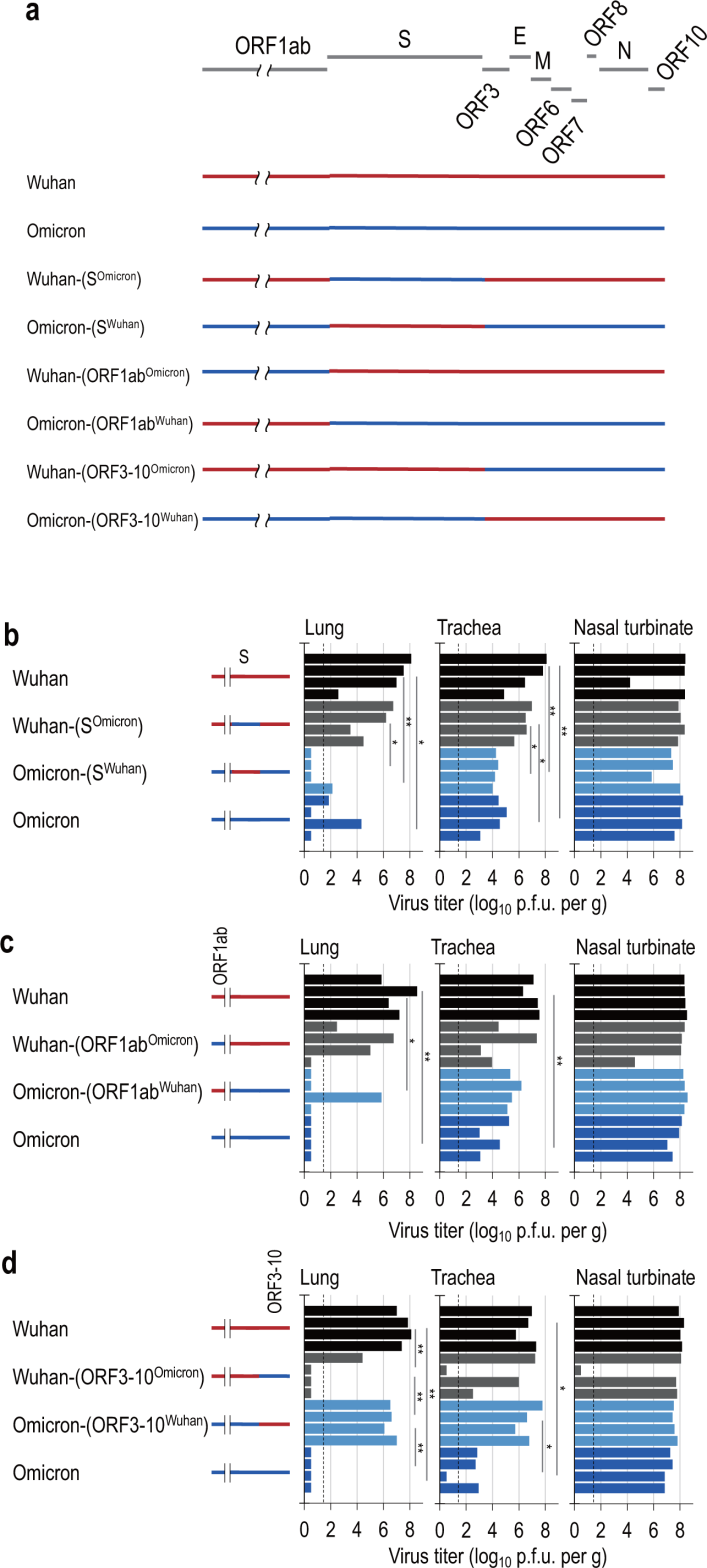
Comparison of the spread of chimeric Wuhan and Omicron viruses in the lower airways of hamsters. (a) Schematic overview of the generated chimeric viruses derived from the Wuhan and Omicron strains. (b–d) Virus titers in the organs of hamsters infected with each chimeric virus and the parent strains [(b) S gene chimeric virus; (c), ORF1ab gene chimeric virus; and (d) ORF3-10 chimeric virus]. For virus titration, four hamsters per group were euthanized 3-days post-infection. Virus titers in the nasal turbinate, trachea, and lungs were determined by means of plaque assays in VeroE6/TMPRSS2 cells. Vertical bars show the average virus titers. Detection limits are indicated by dotted lines. Mean values were compared by an analysis of variance, followed by Tukey’s test (*P < 0.05 and **P < 0.01).

Previous studies have demonstrated an important contribution from the S gene of the Omicron strain in reducing the pathogenicity of SARS-CoV-2 (28). To investigate whether the S gene is involved in the limited replication of Omicron in the lower respiratory tract, we generated chimeric viruses possessing the Omicron S gene in the genetic background of the Wuhan strain and *vice versa* and repeated our experiments using these chimeric viruses (Fig. 3a).

No significant weight changes were observed in hamsters infected with any recombinant viruses (Fig. S3a). We then compared the virus titers of recombinant Wuhan-(S^Omicron^), Omicron-(S^Wuhan^), Wuhan, and Omicron in hamster organs. All recombinant viruses were detected in the nasal turbinate and trachea at 3 and 5 dpi (Fig. 3b; Fig. S3d), whereas Wuhan and Wuhan-(S^Omicron^) were recovered from the lungs of all infected animals on 3 dpi, Omicron and Omicron-(S^Wuhan^) were recovered from the lungs of two of four and one of four infected hamsters on 3 dpi (Fig. 3b). At 5 dpi, virus titers in the lungs of Wuhan-infected hamsters were higher than those in the lungs of hamsters infected with Omicron, Wuhan-(S^Omicron^), or Omicron-(S^Wuhan^) (Fig. S3d). The results show that viral genes other than S promote the optimal spread of SARS-CoV-2 in the lower airways of hamsters.

### The ORF1ab genes of the Omicron strain are not primarily responsible for the limited replication of SARS-CoV-2 in the lower respiratory tract of hamsters

To investigate whether the ORF1ab gene is involved in facilitating optimal Omicron spread to the lower respiratory tract, we generated chimeric viruses possessing the Omicron-ORF1ab gene in the Wuhan strain genetic background and *vice versa* and repeated our experiments using these chimeric viruses (Fig. 3a).

No significant weight changes were observed in hamsters infected with any recombinant viruses (Fig. S3b). We compared the growth properties of recombinant Wuhan-(ORF1ab^Omicron^), Omicron-(ORF1ab^Wuhan^), Wuhan, and Omicron in hamsters intranasally infected with 10^3^ PFU (in 3 µL) of each virus. On 3 and 5 dpi, all recombinant viruses replicated well in the nasal turbinate and trachea (Fig. 3c; Fig. S3e). While the Wuhan virus was recovered from the lungs of all infected hamsters at 3 dpi, Omicron, Omicron-(ORF1ab^Wuhan^), and Wuhan-(ORF1ab^Omicron^) were recovered from the lungs of zero of four, one of four, and three of four infected hamsters, respectively, at 3 dpi (Fig. 3c). At 5 dpi, the virus titers in the lungs of hamsters infected with the Wuhan, Wuhan-(ORF1ab^Omicron^), or Omicron-(ORF1ab^Wuhan^) viruses were higher than those of Omicron-infected hamsters (Fig. S3e). These results suggest that the Wuhan-derived ORF1ab gene is not the primary factor promoting the optimal spread of SARS-CoV-2 in the lower respiratory tract of hamsters.

### Gene regions downstream of ORF3a derived from the Omicron strain limit the replication of SARS-CoV-2 in the lower respiratory tract of hamsters

To investigate whether the genes downstream of the ORF3a gene are involved in facilitating optimal virus spread to the lower respiratory tract, we generated chimeric viruses possessing the Omicron-(genes downstream from ORF3a gene) genes in the Wuhan strain genetic background and *vice versa* and repeated our experiments using these chimeric viruses (Fig. 3a).

No significant weight changes were observed in hamsters infected with any recombinant viruses (Fig. S3c). We compared the titers of recombinant Wuhan-(ORF3-10^Omicron^), Omicron-(ORF3-10^Wuhan^), Wuhan, and Omicron in hamster organs. On 3 and 5 dpi, all recombinant viruses were detected in the nasal turbinate and trachea, except for one hamster infected with Wuhan-(ORF3-10^Omicron^) (Fig. 3d; Fig. S3f). While the Wuhan and Omicron-(ORF3-10^Wuhan^) viruses were recovered from the lungs of all infected animals on day 3 pi, Omicron and Wuhan-(ORF3-10^Omicron^) were recovered from the lungs of zero of four and one of four infected hamsters, respectively, on 3 dpi (Fig. 3d). At 5 dpi, the virus titers in the lungs of hamsters infected with the Wuhan strain were higher than those in the lungs of hamsters infected with Omicron, Omicron-(ORF3-10^Wuhan^), or Wuhan-(ORF3-10^Omicron^) (Fig. S3f). These results suggest that genes downstream of the ORF3a gene derived from the Wuhan virus promote the optimal spread of SARS-CoV-2 in the lower airways of hamsters.

### Various amino acids encoded by genes downstream of ORF3a were under selection pressure during the SARS-CoV-2 epidemic

Downstream of ORF3a, there are numerous ORFs encoding three structural proteins (E, M, and N), as well as genes encoding accessory proteins (ORF3a, ORF3b, ORF3c, ORF3d, ORF6, ORF7a, ORF7b, ORF8, and ORF10), not all of which have been experimentally confirmed (29–32). We compared the amino acid substitutions in the genes from ORF3a to ORF10 between the generated recombinant viruses (Table 1). There were amino acid differences in the ORF3d, E, M, ORF8, N, and ORF10 genes between the generated recombinant Wuhan strain and the Omicron strain. Recombinant SARS-CoV-2 viruses with the ORF3d, E, M, ORF8, N, or ORF10 gene solely from Omicron in the Wuhan genetic background were generated and examined (note that we did not examine the ORF6 and ORF7 genes because there were no amino acid differences between these genes of the generated recombinant Wuhan and Omicron strains). However, the phenotypes of these viruses did not change from those of the parental Wuhan strain (see supplemental material), suggesting that the simultaneous presence of multiple mutations may affect the restriction of virus spread to the lungs. In addition, we compared the amino acid substitutions in the genes from ORF3a to ORF10 between the generated recombinant viruses and some variants (Table 1). We found amino acid substitutions (ORF3d-P21S, E-T9I, M-Q19E, M-A63T, and N-31-33del) that were unique to various Omicron strains (BA. 1, BA. 2, BA4, or BA. 5). For ORF3d, stop codons were found in the ORF in some strains, suggesting that these strains may not produce functional proteins.

**TABLE 1:** Comparison of the amino acid substitutions in the gene regions downstream of ORF3a between the generated recombinant viruses and various variants^*a*^^,^^*b*^

Virus	GISAID ID	ORF3a	ORF3d	E	M	ORF6	ORF7a	ORF7b	ORF8	N	ORF10
Recombinant (Wuhan)	This study	–	–	–	–	–	–	–	–	–	–
Recombinant (Omicron)	This study	–	**P21S**	**T9I**	D3G, **Q19E**, **A63T**	–	–	–	S84L	P13L, **31–33del**, R203K, G204R	V30L
Wuhan	EPI_ISL_402124	–	–	–	–	–	–	–	S84L	–	–
Omicron-BA.1	EPI_ISL_8185458	–	**P21S**	**T9I**	D3G, **Q19E**, **A63T**	–	–	–	S84L	P13L, **31–33del**, R203K, G204R	–
Omicron-BA.2	EPI_ISL_8135710	T223I	**P21S**	**T9I**	**Q19E**, **A63T**	D62L	–	–	S84L	P13L, **31–33del**, R203K, G204R, S413R	–
Omicron-BA.4	EPI_ISL_11542550	T223I	**P21S**	**T9I**	**Q19E**, **A63T**	D62L	–	LF11	S84L	P13L, **31–33del**, P151S, R203K, G204R, S413R	–
Omicron-BA.5	EPI_ISL_11542604	T223I	**P21S**	**T9I**	D3N, **Q19E**, **A63T**	–	–	–	S84L	P13L, **31–33del**, E136D, R203K, G204R, S413R	–
Delta	EPI_ISL_1758376	S26L	–	–	I82T	–	V82A, T120I	T40I	S84L, 119–120del	D63G, R203M, G215C, D377Y	–
Alpha	EPI_ISL_674612	–	–	–	–	–	–	–	27–121del	D3L, R203K, G204R, S235F	–
Beta	EPI_ISL_940877	Q57H, S171L	14stop	P71L	–	–	–	–	S84L	T205I	–
Gamma	EPI_ISL_2777382	S253P	–	–	–	–	–	–	S84L, E92K	P80R, R203K, G204R	–
Lambda	EPI_ISL_1138413	–	–	–	–	–	–	–	S84L	P13L, R203K, G204R, G214C, T366I	–
Mu	EPI_ISL_2086281	Q57H, VNP256-258IQstop	14stop	–	–	–	–	–	T11K, P38S, S67F, S84L	T205I	–
Eta	EPI_ISL_760883	–	–	L21F	I82T	2del	–	–	S84L	SD2-3Y, A12G, T205I	–
Iota	EPI_ISL_1300881	P42L, Q57H	14stop	–	–	–	–	–	T11I, S84L	P199L, M234I	–
Theta	EPI_ISL_1660475	–	–	–	–	–	–	–	K2Q, S84L	R203K, G204R	–
Kappa	EPI_ISL_1384866	S26L	–	–	I82S	–	V82A	–	S84L	R203M, D377Y	–
Zeta	EPI_ISL_717936	–	–	–	–	–	–	–	S84L	A119S, R203K, G204R, M234I	–
Epsilon-B1.429	EPI_ISL_648527	Q57H, A99V	14stop	–	–	–	–	–	S84L	T205I	–
Epsilon-B1.427	EPI_ISL_1531902	Q57H	14stop	–	–	–	–	–	S84L	T205I	–

^*a*^ Boldface type indicates amino acid substitutions that were unique to various Omicron strains.

^*b*^ –, no mutation was detected compared to the sequences from the recombinant Wuhan strain.

To examine whether natural selection has affected the E, M, N, or ORF10 genes, we first generated a phylogenetic tree of selected 575 SARS-CoV-2 genomes that passed quality control (see Materials and Methods section for details). The obtained phylogenetic tree showed the same topology reported previously (Fig. S4) (33). The possibility of positive selection on each codon site was examined (dN/dS > 1) by using CODEML and SLAC (Table 2, see also the Materials and Methods section). Note, we did not examine selection on the ORF3d gene because it overlaps with the ORF3a gene. We found that the E and M genes were under statistically significant selection based on the likelihood ratio test of the CODEML analysis (*P*-value = 0.0049 and 2.8 × 10^−6^ for E and M, respectively, see Materials and Methods). We identified that the codon at position 9 of E and the codons at positions 3 and 19 of M are under positive selection. The SLAC analysis of the E, M, and N genes indicated that only position 3 of M was under statistically significant positive selection. These results indicate that various amino acids in non-S genes are under selection and have acquired various functions. We further examined those amino acid sites in 60 representative sarbecoviruses, including SARS-CoV-2-related bat coronaviruses, and found that no bat sarbecoviruses had Omicron-type amino acids or deletions (Fig. S5). These results suggest that some Omicron-like amino acid substitutions, particularly in the E, M, and N genes, might be adaptive mutations for humans that were acquired during the SARS-CoV-2 epidemic.

**TABLE 2:** Positive selection analysis^*a*^^,^^*b*^

Gene	Codon position	Reference	Alternative	CODEML (posterior probability)	SLAC (*P*-value)
E	9	T	I	0.998	0.409
M	3	D	G	1	0.059
M	19	Q	E	0.999	0.244
M	63	A	T	–	0.667

^*a*^ The possibility of positive selection on each codon site of the E, M, N, and ORF10 genes was examined by using CODEML and SLAC.

^*b*^ –, the posterior probability was not significant (<0.95).

## DISCUSSION

The Omicron strain of SARS-CoV-2 has spread worldwide since its emergence in late 2021, and its sub-lineages are now becoming established in human society (11). Compared to previous strains, the Omicron strain is less invasive in the lungs and other lower respiratory tracts and causes less severe disease (12, 13). One reason for this is that humans are acquiring immunity through previous infection and vaccination (34, 35). There are also reports that the virus itself has changed, becoming attenuated (14, 16, 17). Using the low-volume inoculation system established in this study, which reflects natural human infection, we showed that the Omicron strain spreads less efficiently into the lungs of hamsters compared with the Wuhan strain. To identify the viral gene region responsible for this lower respiratory tract restriction, we generated chimeric viruses with Omicron genes in the Wuhan strain genetic background and *vice versa* and examined the efficiency of virus spread in the respiratory tracts of infected hamsters. We found that viral genes downstream of ORF3a, but not the S gene, were responsible for replication restriction in the lower respiratory tract of hamsters infected with the Omicron strain. Furthermore, molecular evolutionary analysis of SARS-CoV-2 revealed positive selection of genes downstream of ORF3a (i.e., M and E genes). Our findings provide insight into the adaptive evolution of the virus in humans during the pandemic convergence phase.

Studies using *in vivo* and *ex vivo* models have shown that Omicron strains grow efficiently in cells of the upper airway but are less likely to grow in lung cells (14–17). Suzuki et al. (14) speculated that the cell fusion activity of the S protein of Omicron strains is lower than that of conventional D614G-bearing strains (i.e., B.1.1 lineage) and Delta strains, which may reduce their infection efficiency. Other reports suggest that the decreased binding affinity of the S protein of Omicron to human ACE2 may be responsible for its decreased infection efficiency (36). However, studies using nasal epithelial organoid cultures have reported that SARS-CoV-2 adheres to motor cilia via the ACE2 receptor and that the S protein of Omicron strains binds to these cilia with higher affinity than conventional strains (i.e., D614G-bearing strains and Delta strains), facilitating virus entry (37). Thus, the relevance of the binding affinity of the S protein for human ACE2 to the virus infection efficiency remains controversial. It may be that the amino acid substitutions in the S protein of the Omicron strain reduce the efficiency of TMPRSS2 cleavage by the S protein, resulting in less efficient intracellular entry via TMPRSS2 and reducing the likelihood of infection of TMPRSS2-expressing lung cells (28, 38, 39). As for the possibility that factors other than S may be involved, Chen et al. (40) identified nonstructural protein 6 as a factor in determining the attenuated Omicron phenotype in their studies using K18-hACE2 mice. In the present study, we found that the S gene is not involved in suppressing the spread of the Omicron strain of the virus into the lungs, but genes downstream of ORF3a are involved. Our findings likely differ from those of previous reports (14, 16, 17) probably because we used hamsters rather than K18-hACE2 mice and a low-volume inoculation system that reflects natural infection in humans. Our data suggest that there were amino acid differences in the ORF3d, E, M, ORF8, N, and ORF10 genes between the generated recombinant Wuhan strain and the Omicron strain, and several genes, rather than a single gene, downstream of ORF3a may be associated with the limited replication of SARS-CoV-2 in the lower respiratory tract of hamsters since none of the recombinant SARS-CoV-2 viruses possessing solely the ORF3d, E, M, ORF8, N, or ORF10 gene of Omicron in the Wuhan genetic background showed limited spread to the lungs (Fig. S6 and S7). The known functions of these proteins are as follows: E regulates viral envelope fluidity, M plays a central role in viral assembly, ORF8 suppresses MHC class I function and cytotoxic T cell-mediated immune responses, N packages the viral genome into the ribonucleocapsid complex, and ORF10 suppresses innate immunity (32, 41, 42). In addition, Omicron M and E decrease the efficiency of particle formation (43), which may contribute to the decreased infection efficiency of these strains.

We found that the degree of cilia damage in the trachea of infected hamsters differed between the Wuhan and Omicron strains (Fig. 2d). The airway lumen is covered by multiciliated epithelial cells with 200–300 cilia, forming an epithelial sheet. These airway cilia generate mucus flow through coordinated circling movements and serve as a mucociliary clearance mechanism that expels pathogens (44, 45). Therefore, damage to airway cilia is thought to impair ciliary function and facilitate pathogen entry from the upper airways into the lower airways. In the trachea and lungs of SARS-CoV-2-infected patients, ciliated cells have been observed to drop out into the airway lumen, exposing basal cells (46). In the present study, the Wuhan strain replicated well in the hamster trachea and severely damaged the airway cilia, consistent with a previous report (27). Mucociliary clearance by the cilia was reduced, allowing the virus to reach and multiply in the lungs. In contrast, the Omicron strain replicated less efficiently in the trachea, and there was almost no damage to the airway cilia; therefore, the virus could have been eliminated from the trachea to the upper lungs by mucus cilia movement. Less damage to the airway cilia, therefore, may have contributed to limiting the spread of the Omicron strain to the lungs, but more detailed analysis is needed to elucidate the mechanism.

Tracking and understanding the evolutionary process of RNA viruses may help us predict how RNA viruses will evolve in the future. Natural selection “fixes in” favorable mutations that confer high infectivity, for example, the D614G mutation in the S protein of SARS-CoV-2 (47). In addition, many mutations are lost due to severe bottlenecks that occur during infection, whereas other mutations are passed on accidentally without selective advantage (48). In the present study, our molecular evolutionary analysis revealed a positive selection of genes downstream of ORF3a (M and E genes). However, since the S protein is a major driver of the genomic evolution of SARS-CoV-2 (49), we cannot rule out the possibility that the positively selected amino acids detected were due to a hitch-hiking effect of the S protein (50). Given that positively selected sites in M and E were not found in other SARS-related bat coronaviruses, it is possible that the M and E mutations are the result of the adaptive evolution of SARS-CoV-2 in humans. It is possible that other genes besides E and M are involved in the adaptive evolution to humans, but the deletion of 31–33 amino acids in the N protein was not analyzed in this study because the dN/dS ratio could not be calculated. ORF3d may also be involved in the antibody response (51), but due to the overlap of various genes (52) and the complexity of its genetic structure, evolutionary analysis was not performed in this study. Further studies are needed to investigate the evolutionary pressures acting on these genomic changes.

## MATERIALS AND METHODS

### Cells

VeroE6/TMPRSS2 (JCRB 1819) cells were maintained in Dulbecco’s modified Eagle’s medium (DMEM) supplemented with 10% fetal bovine serum (FBS), 1% penicillin-streptomycin solution, and 1 mg/mL geneticin (G418; Nacalai Tesque, Cat# 09380-44). HEK293-hACE2/hTMPRSS2 cells (53) were maintained in DMEM supplemented with 10% FBS and 1% penicillin-streptomycin solution. VeroE6/TMPRSS2 and HEK293-hACE2/hTMPRSS2 cells were maintained at 37°C with 5% CO_2_.

### Biosafety statement

All procedures were performed in an enhanced biosafety level 3 (BSL3) containment laboratory approved for such use by Osaka University. The experimental plans, including the generation of recombinant chimeric viruses, were reviewed and approved by the Research Microbiology Safety Committee of the Research Institute for Microbial Diseases, Osaka University. All personnel completed rigorous biosafety, biosecurity, and BSL3 training before participating in BSL3-level experiments. Personal protective equipments, including Tyvek suits, disposable gowns, shoe covers, hoods, double-layered gloves, face shields, and N95 masks, were used. The researchers involved in working with recombinant chimeric viruses received at least two booster shots of the SARS-CoV-2 mRNA vaccine before beginning the research. All hamsters were housed in individually ventilated cages (IsoCages; Techniplast) in enhanced BSL3 containment laboratories at Osaka University. All *in vivo* and *in vitro* experiments were carried out in class 2 biosafety cabinets maintained under negative pressure.

### Reverse genetics

Recombinant SARS-CoV-2 was generated by using the circular polymerase extension reaction (CPER) as previously described (54). Briefly, nine DNA fragments encoding the partial genome of the SARS-CoV-2 Wuhan strain (hCoV-19/Japan/TY-WK-521/2020, GISAID ID: EPI_ISL_408667) or the SARS-CoV-2 Omicron strain (hCoV-19/Japan/TY38-873/2021, GISAID ID: EPI_ISL_7418017) were amplified by PCR using PrimeSTAR GXL DNA polymerase (Takara, Cat# R050A). A linker fragment encoding hepatitis delta virus ribozyme, bovine growth hormone poly A signal, and cytomegalovirus promoter was also amplified by PCR. The 10 DNA fragments were then mixed and used for CPER (54).

To produce recombinant SARS-CoV-2 (seed virus), the CPER products were transfected into HEK293-hACE2/hTMPRSS2 cells using TransIT-LT1 (Takara, Cat# MIR2305). At 1-day post-transfection, the culture medium was replaced with DMEM (high glucose) (Nacalai Tesque, Cat# 08459-64) containing 2% FBS and 1% penicillin-streptomycin solution. At 4–10 days post-transfection, the culture medium was collected and stored at −80°C. The viruses were used to inoculate VeroE6/TMPRSS2 cells to grow viruses for stock viruses. Stock viruses were sequenced, and amino acid substitutions in each virus are summarized in Table S2. All experiments with transfectants generated by reverse genetics were performed in an enhanced biosafety level 3 containment laboratory approved for such use by Osaka University.

### Animal experiments

All animals were housed under specific pathogen-free conditions in a temperature and humidity-controlled environment with a 12 h:12 h light:dark cycle and *ad libitum* access to water and standard laboratory chow. Virus inoculations were performed under anesthesia, and all efforts were made to minimize animal suffering. *In vivo* studies were not blinded, and animals were randomly assigned to infection groups. No sample-size calculations were performed to power each study. Instead, the sample size was determined based on prior *in vivo* virus challenge experiments.

### Experimental infection of Syrian hamsters

Seven-week-old male wild-type Syrian hamsters (Japan SLC Inc., Shizuoka, Japan) were used in this study. Baseline body weights were measured before infection. Under isoflurane anesthesia, three or four hamsters per group were intranasally inoculated with 10^3^ PFU in 3 µL or 10^3^ PFU in 30 µL of recombinant virus. Body weight was monitored daily after infection. For virological and pathological examinations, three or four hamsters per group were intranasally infected with recombinant viruses; at 1, 3, 5, and 7 dpi, the animals were euthanized, and the nasal turbinate, trachea, and lungs were collected. The virus titers in the nasal turbinate, trachea, and lungs were determined by using plaque assay on VeroE6/TMPRSS2 cells.

### Histopathology and immunohistochemistry

Excised animal tissues were fixed in 10% Formalin Neutral Buffer Solution (FUJIFILM Wako Pure Chemical Corporation, Cat#060-01663) and processed for paraffin embedding. Nasal turbinate tissues were decalcified with 14% EDTA (pH 7.2) prior to paraffin embedding. Tissue sections were stained with HE. To detect SARS-CoV-2 antigen, sections were pretreated with heat-induced epitope retrieval in 10 mM citrate buffer (pH 6.0) and then immunohistochemistry was performed using a rabbit monoclonal antibody against SARS-CoV-2 nucleocapsid protein (1:1,000, GeneTex, clone HL344). Antigens were visualized with a detection reagent and diaminobenzidine (Nichirei, N-Histofine Simple Stain Mouse MAX PO).

### Scanning electron microscopy

Hamster tracheal samples were fixed with 2% glutaraldehyde in 0.1 M phosphate buffer overnight at 4°C. Samples were then washed in 0.1 M phosphate buffer twice and processed by alternating incubations in 1% osmium tetroxide. After dehydration by incubation in increasing concentrations of ethanol, the samples were t-butyl alcohol freeze-dried, osmium-coated, mounted on a stub, and analyzed by field emission scanning electron microscopy (Hitachi S-4800).

### Evolutionary analysis

SARS-CoV-2 genome and annotation information used in this study was downloaded from the GISAID database (https://www.gisaid.org) on 12 January 2023 (14,295,625 sequences). We collected genomes matching the following criteria: (i) isolated from humans; (ii) having a sampling date between 1 January 2021 and 31 December 2022; (iii) having less than 300 undetermined nucleotides in the genome; and (iv) differing by less than 300 nucleotides compared with the reference SARS-CoV-2 genome, Wuhan-Hu-1 (EPI_ISL_402125). By using these criteria, we obtained 9,415,793 genomes. From them, we randomly sampled five genomes per month per continent (Africa, North America, South America, Asia, Europe, and Oceania); 720 genomes were thus selected. We also added the reference strain Wuhan-Hu-1 as a data set. To remove possible recombinant variants that might affect the selection analysis, RDP4 version 4.101 (55) with default parameters was applied multiple times. After this step, 679 recombination-free SARS-CoV-2 genomes (including Wuhan-Hu-1) remained. Since we aimed to analyze the genes of ORF3d, E, M, N, and ORF10, any genomes containing any undetermined sites in those genes were also excluded. Finally, 575 SARS-CoV-2 genomes, including the Wuhan-Hu-1 genome and EPI_ISL_7418017, which were used for experimental analyses, were selected for the selection test. A list of the GISAID IDs used in this study is available at https://epicov.org/epi3/epi_set/230411xq. With the 575 SARS-CoV-2 genomes, we generated a multiple alignment using the FFT-NS-1 program in MAFFT suite version 7.407 (56). Gaps in the multiple alignment were removed by referring to the genomic locations in the Wuhan-Hu-1 genome. Based on the multiple sequence alignment, a maximum likelihood-based tree was constructed using IQ-TREE 2 version 2.1.3 with -B 1000 -T AUTO options (57). The phylogenetic trees were visualized by using ggtree v2.4.1 (58).

To infer natural selection on each codon site of the E, M, N, and ORF10 genes, we applied the CODEML program in the PAML software package version 4.9j (59) and the SLAC program (60) via the Datamonkey website (61) accessed on 11 April 2023. For CODEML, we compared the likelihoods of M7 and M8 site models to test for positive selection by using the χ^2^ distribution with a likelihood ratio test (LRT), degree of freedom = 2. When LRTs indicated positive selection (*P* ≤ 0.05), we examined the posterior probability based on the Bayes empirical Bayes (BEB) methods and determined that they were under positive selection with a BEB of ≥0.95. For SLAC, amino acid sites showing a *P* value ≤ 0.05 based on LRT were chosen as selective sites.

### Molecular evolutionary phylogenetic analysis and detection of amino acid substitutions

To compute the phylogenetic tree of the whole genome of the 60 representative sarbecoviruses including SARS-CoV-2-related bat coronaviruses, their genome sequences were aligned using the program in MAFFT version 7.453 (56). To estimate the appropriate nucleotide substitution model, we used ModelTest-NG (62) based on the Akaike information criterion values, and GTR + I + G4 was selected. We then computed a phylogenetic tree using RAxML-NG version 1.0.0 (63) with rapid 100 bootstrap replicates (64).

To obtain the E, M, and N genes of the 60 representative coronaviruses, TBLASTN searches were performed against the entire genome sequence using the proteins of the SARS-CoV-2 isolate Wuhan-Hu-1[NC_045512.2; EPI_ISL_402125], as queries with the options *e*-value threshold 1e−20. The results of the TBLASTN analysis were used for manual annotations. To detect amino acid substitutions encoded by the E, M, and N genes of the 60 representative coronaviruses, their amino acid sequences were aligned using the program in MAFFT version 7.453 (56).

### Statistical analysis

All data were analyzed by using GraphPad Prism software (version 9.4.0). Statistical analysis included ANOVA with multiple correction post-tests. Differences among groups were considered significant for *P* values < 0.05.

## Data Availability

The data underlying this article will be shared upon reasonable request to the corresponding author.

## SUPPLEMENTAL MATERIAL

Supplemental figures jvi.01784-23-s0001.pdf Figures S1 to S7.

Supplemental material jvi.01784-23-s0002.docx Supplemental text and legends for Fig. S1 to S7.

Table S1 jvi.01784-23-s0003.xlsx Viral titer and histopathological analysis in organs of recombinant virus-infected hamsters in the high-volume or low-volume infection system.

Table S2 jvi.01784-23-s0004.xlsx Comparison of the amino acid substitutions in each protein in the recombinant viruses in this study.

ASM does not own the copyrights to Supplemental Material that may be linked to, or accessed through, an article. The authors have granted ASM a non-exclusive, world-wide license to publish the Supplemental Material files. Please contact the corresponding author directly for reuse.
