# Supplementary material for: Genes involved in the limited spread of SARS-CoV-2 in the lower respiratory airways of hamsters may be associated with adaptive evolution
Source: J Virol. 2024 Apr 16;98(5):e01784-23. doi: 10.1128/jvi.01784-23 (PMC11092350; doi:10.1128/jvi.01784-23)
Supplement: Supplemental figures — Figures S1 to S7. [file jvi.01784-23-s0001.pdf]

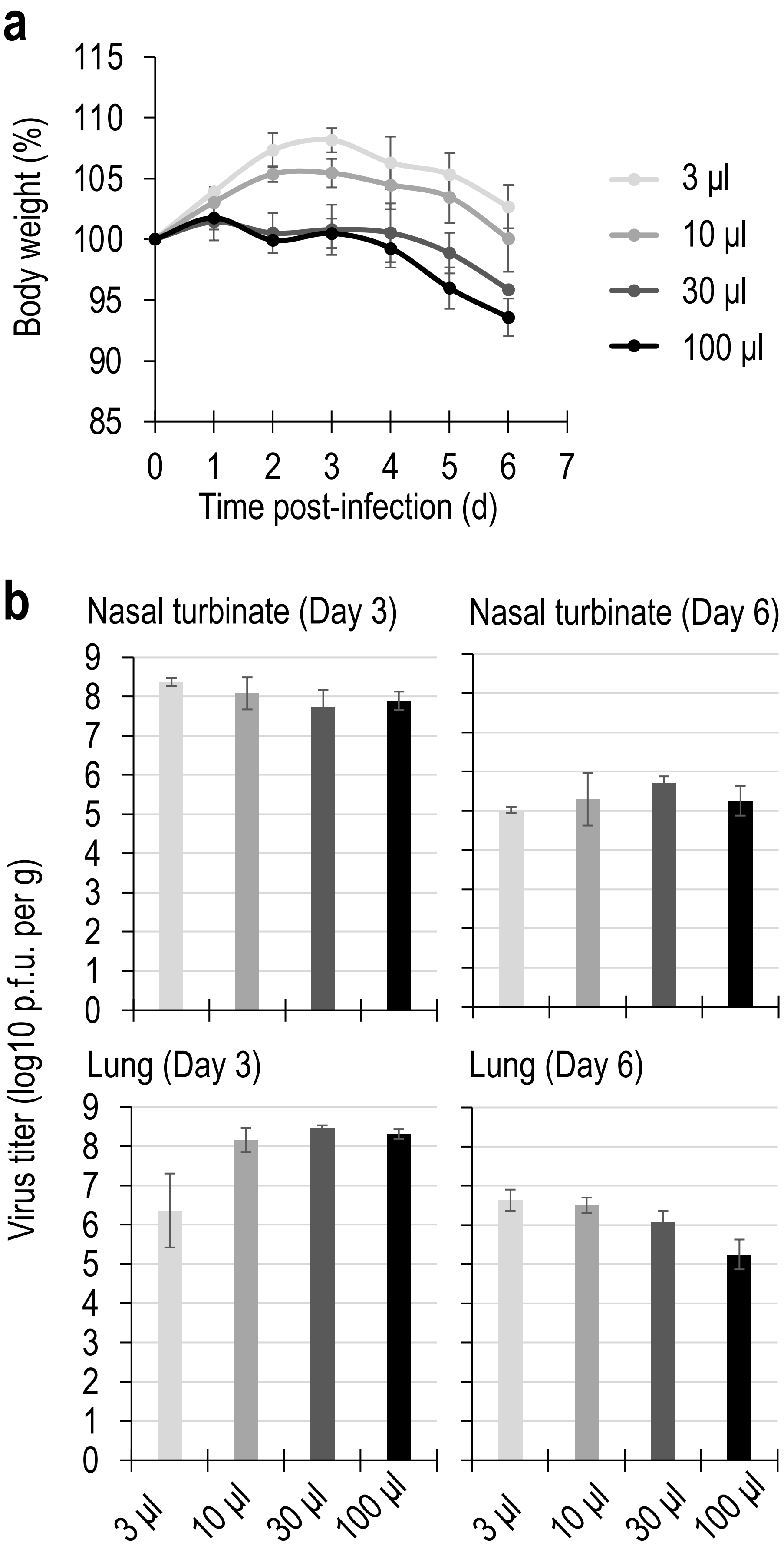

Supplementary Fig. 1

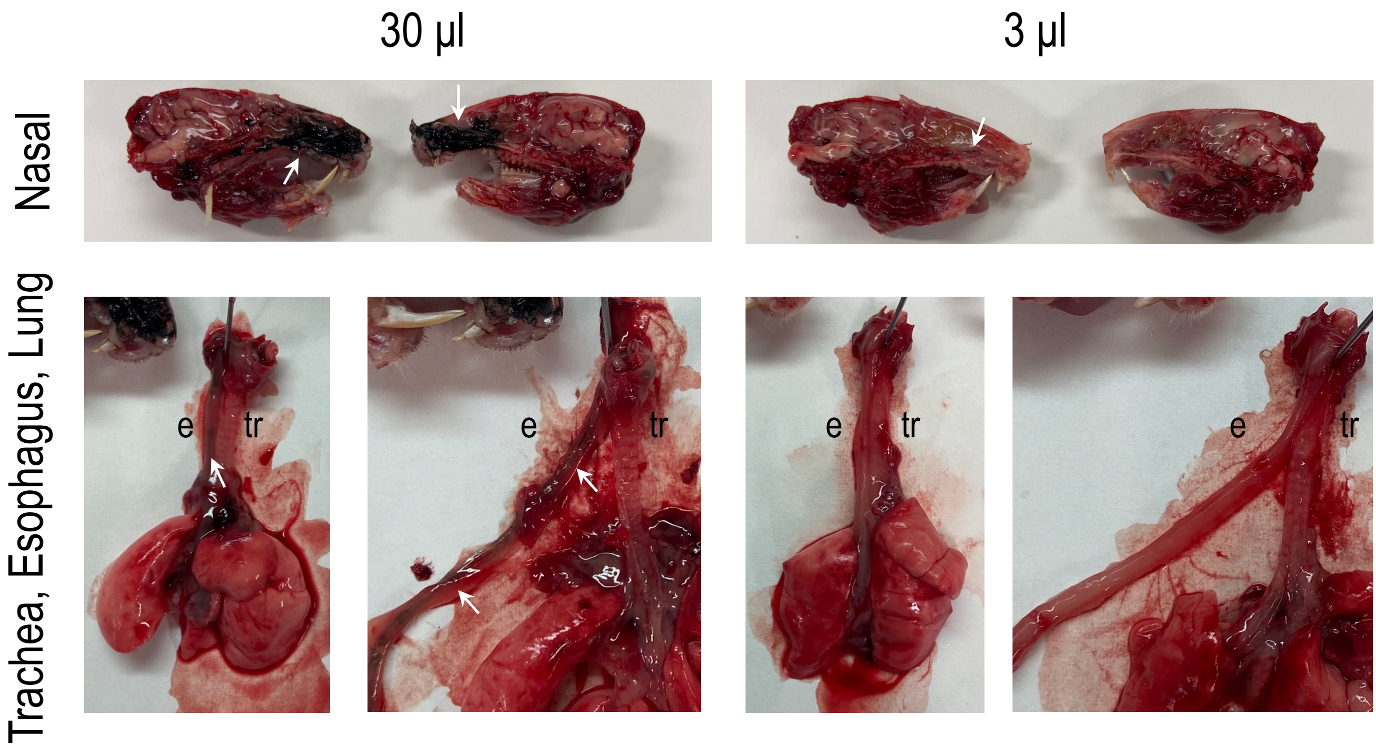

Supplementary Fig. 2

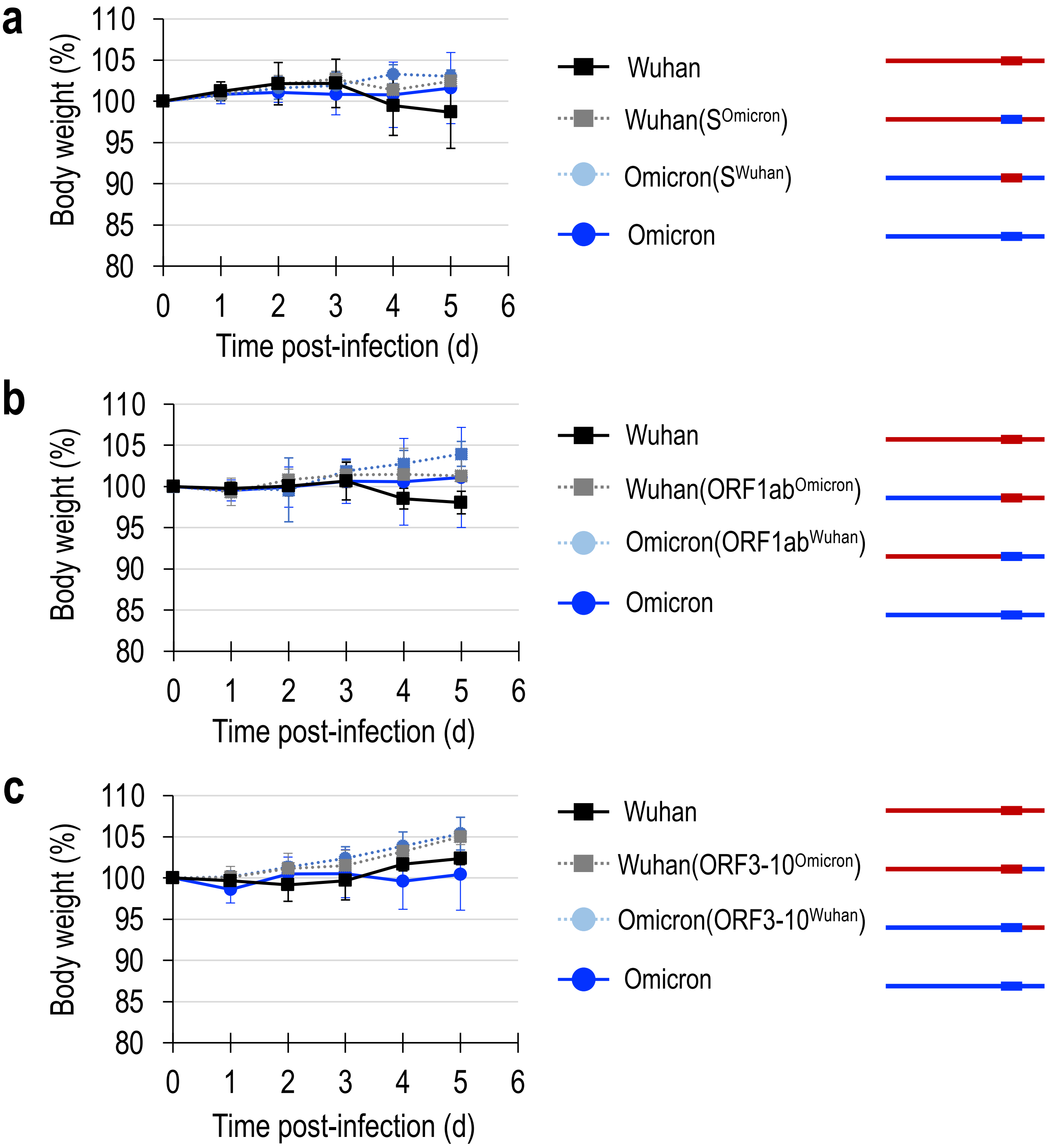

Supplementary Fig. 3

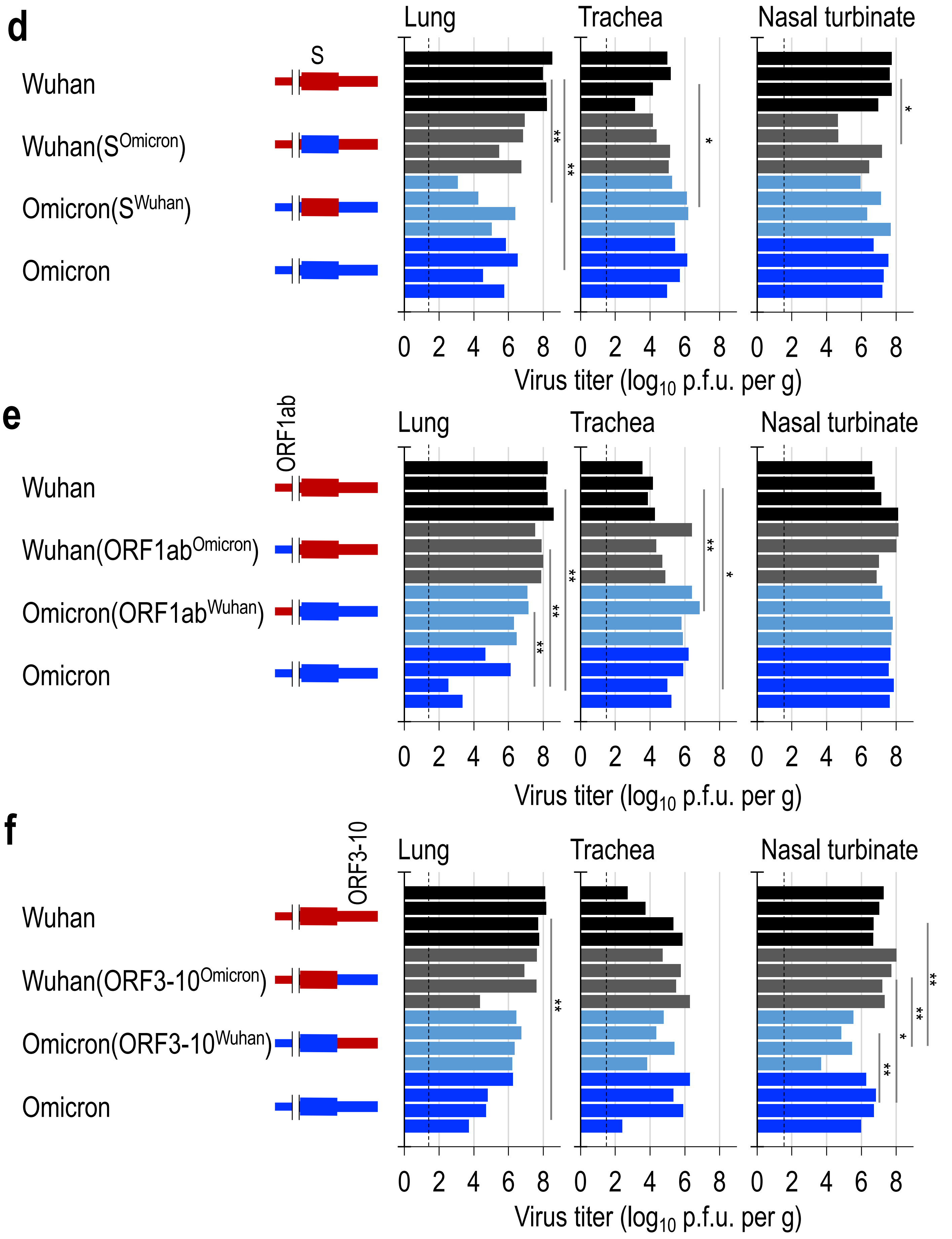

Supplementary Fig. 3

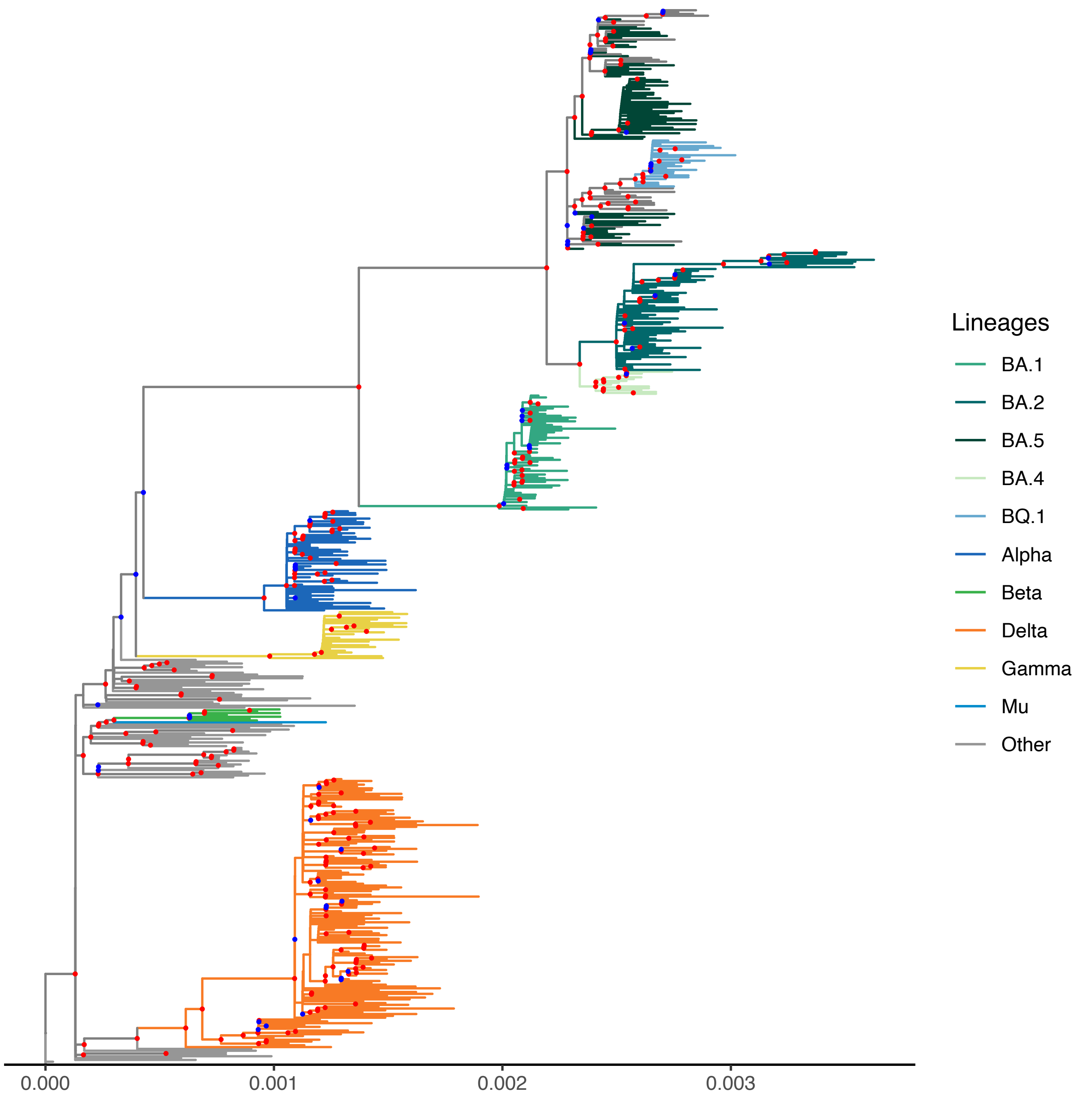

Supplementary Fig. 4

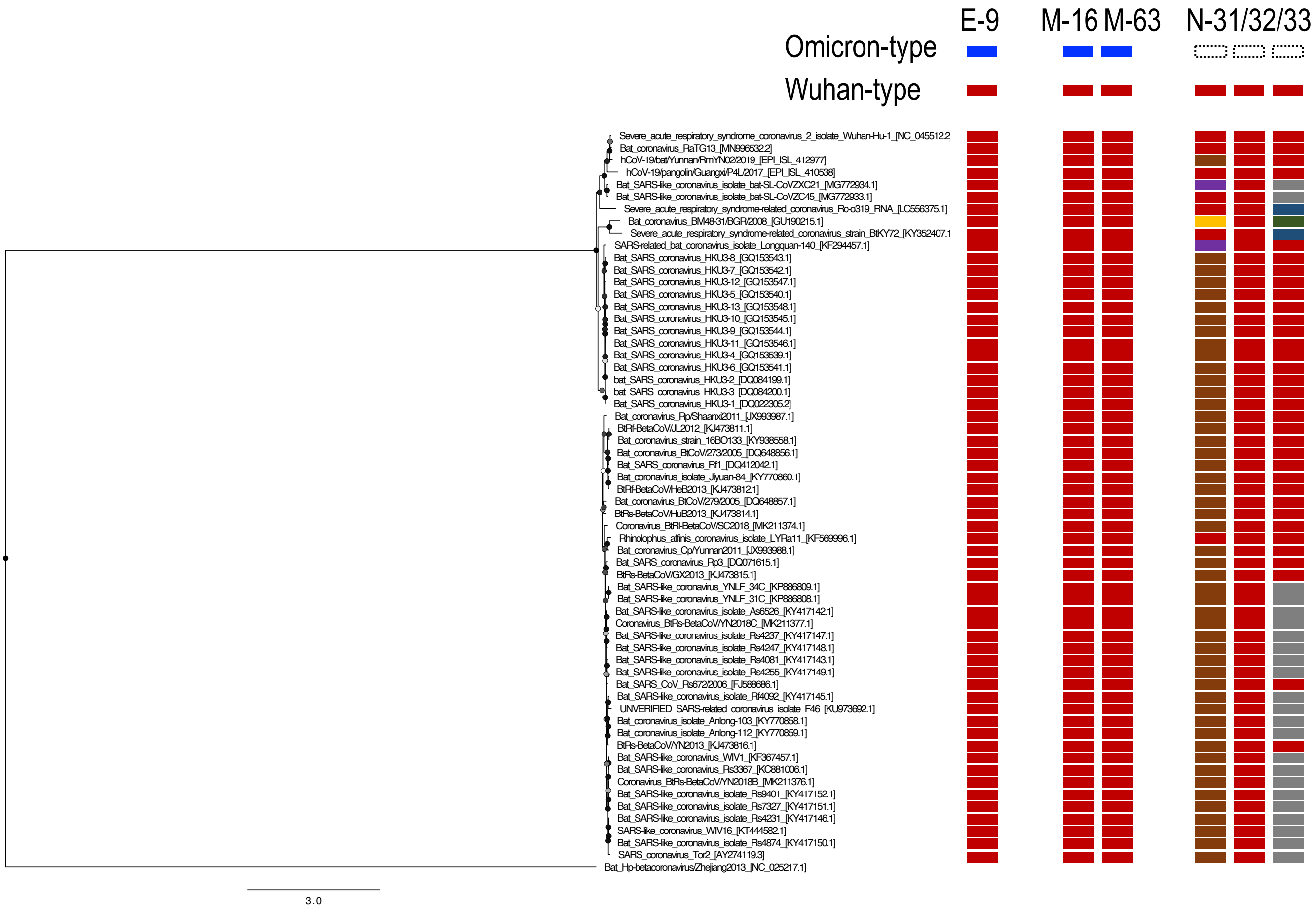

Supplementary Fig. 5

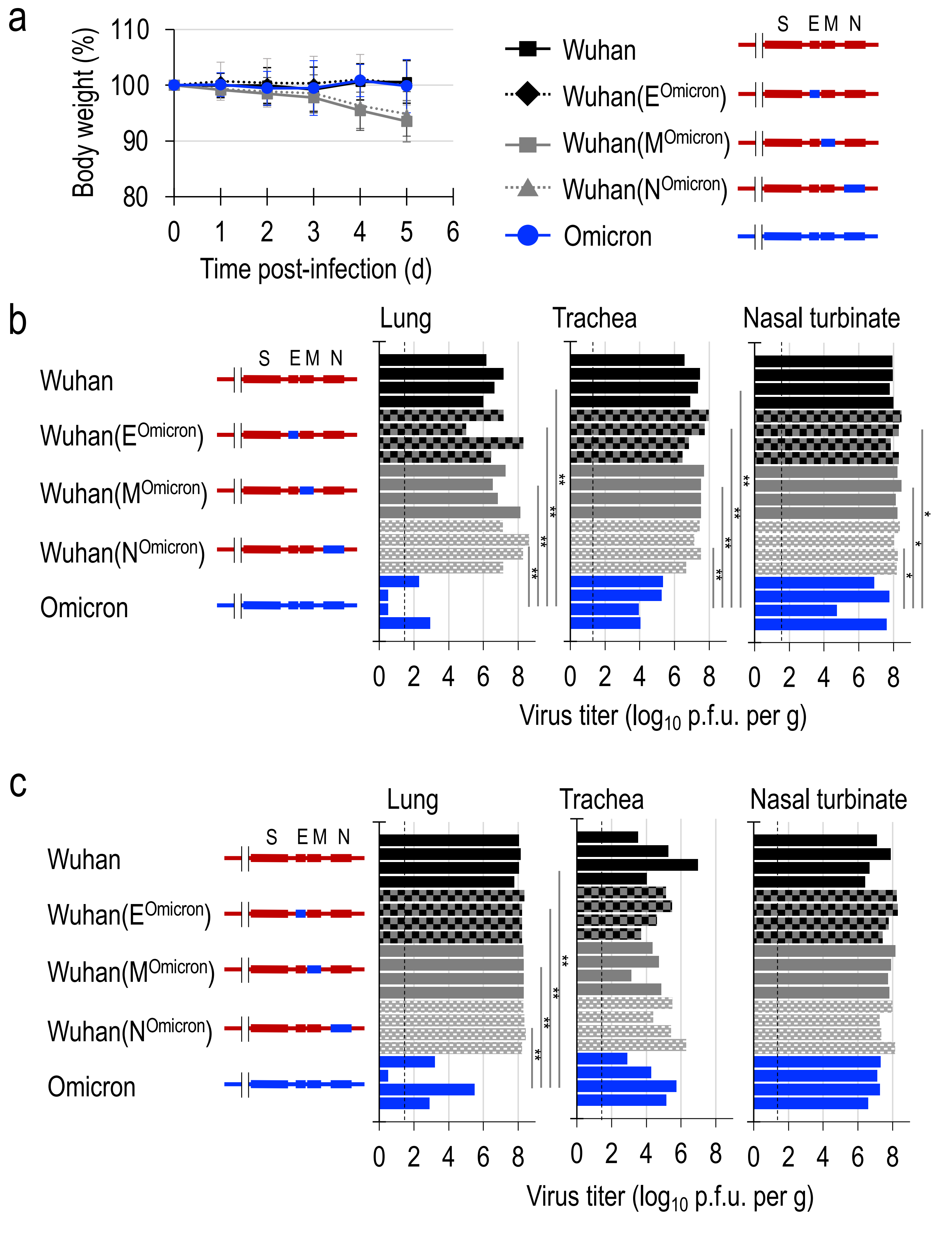

Supplementary Fig. 6

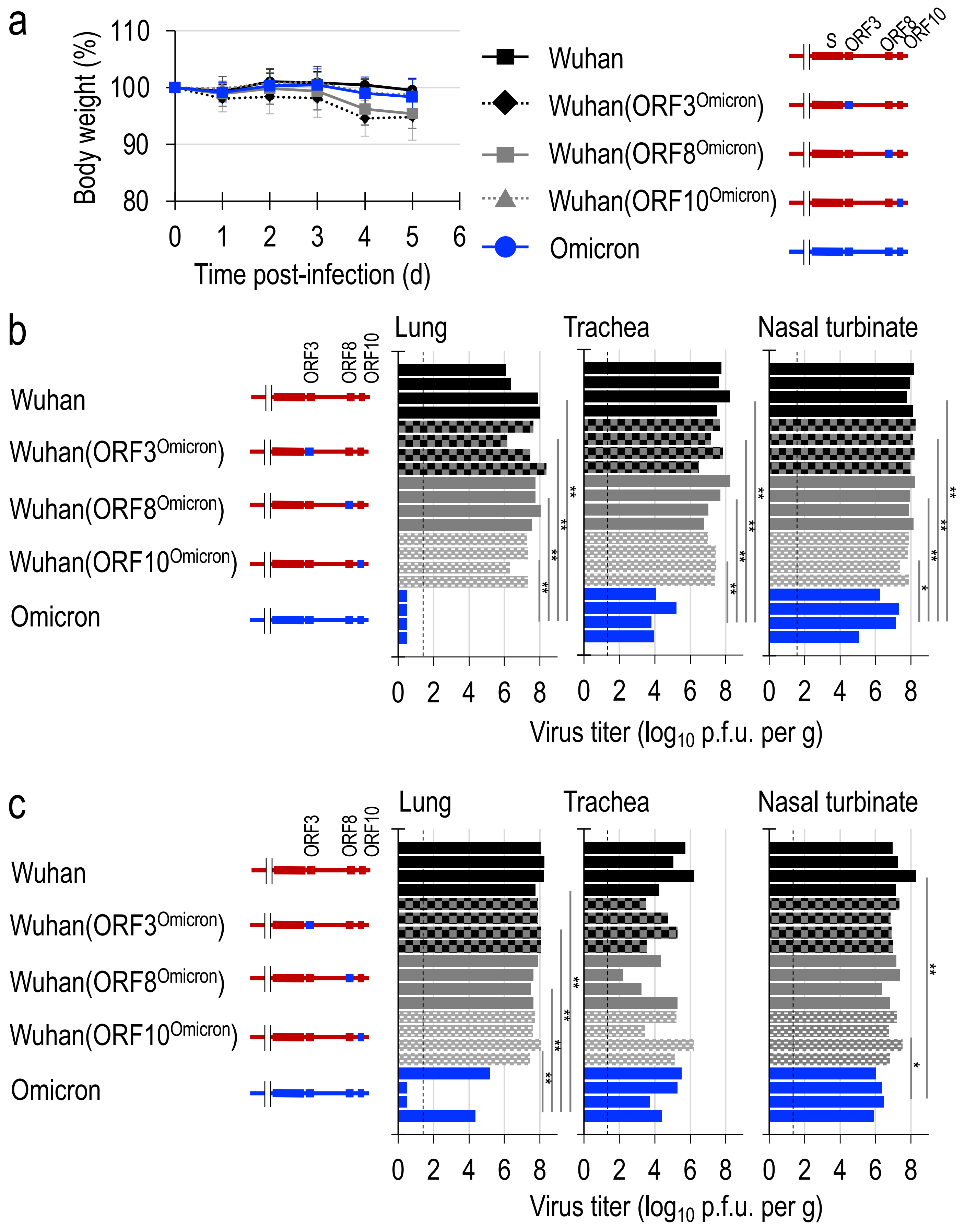

Supplementary Fig. 7
