# Supplementary material for: Genes involved in the limited spread of SARS-CoV-2 in the lower respiratory airways of hamsters may be associated with adaptive evolution
Source: J Virol. 2024 Apr 16;98(5):e01784-23. doi: 10.1128/jvi.01784-23 (PMC11092350; doi:10.1128/jvi.01784-23)
Supplement: Supplemental material — Supplemental text and legends for Fig. S1 to S7. [file jvi.01784-23-s0002.docx]

**Revised manuscript**

**Supplementary information**

**Genes involved in the limited spread of SARS-CoV-2 in the lower respiratory** **airways of hamsters may be associated with adaptive evolution**

Kosuke Takada, Yasuko Orba, Yurie Kida, Jiaqi Wu, Chikako Ono, Yoshiharu Matsuura, So Nakagawa, Hirofumi Sawa, and Tokiko Watanabe.

- Supplementary materials and methods
- Supplementary results
- Supplementary Figures S1–S7
- Supplementary figure legends
- Supplementary Tables S1–S2 (separate Excel files)

**Supplementary materials and methods**

**Experimental infection of Syrian hamsters.**

One-month-old male wild-type Syrian hamsters (Japan SLC Inc., Shizuoka, Japan) were used in this study. Baseline body weights were measured before infection. Under isoflurane anesthesia, three hamsters per group were intranasally inoculated with 10^3^ PFU of Wuhan strain (SARS-CoV-2/UT-NCGM02/Human/2020/Tokyo) in 100, 30, 10, or 3 μl. Body weight was monitored daily after infection. For virological examinations, three hamster per group were intranasally infected with recombinant viruses; 3 and 6 dpi, the animals were euthanized and the nasal turbinate and lungs were collected. The virus titers in the lungs were determined by performing plaque assays on VeroE6/TMPRSS2 cells.

**Tissue-marking dye distribution in hamsters.**

To compare inoculum spread through the airways of hamsters between high-volume inoculation (30 μl) and low-volume inoculation (3 μl), two hamsters were lightly anesthetized by inhalation of isoflurane and inoculated intranasally with either 30 μl or 3 μl of a tissue-marking dye (Polysciences Inc., Warrington, PA). After administration of the tissue-marking dye, the hamsters fully recovered and moved freely around the cage. Approximately 30 min later, the hamsters were anesthetized and humanely euthanized. The entire length of the airway was then dissected and the distribution of the tissue-marking dye was visually assessed.

**Supplementary results**

**Comparison of different infection volume systems in a hamster model using the Wuhan strain.**

To determine the appropriate inoculum volumes for this study, we compared different inoculum volumes of the Wuhan strain in a hamster model. When we administered 100, 30, 10, or 3 μl of virus liquid to hamsters intranasally. We found that weight loss was more severe with higher inoculum volumes (Supplementary Fig. 1a), suggesting the higher the inoculum volume, the higher the virulence in the hamsters.

Next, we examined virus replication in the upper and lower respiratory tracts of the hamsters infected with 100, 30, 10, or 3 μl of virus liquid. We found that the mean virus titers in lungs from the 100-, 30-, 10-, and 3 μl-inoculation groups at 3 dpi were 10^8.31^, 10^8.46^, 10^8.16^, and 10^6.36^ PFU/g, respectively, whereas those of the nasal turbinate for the 100-, 30-, 10-, and 3 μl-inoculation groups at 3 dpi were 10^7.89^, 10^7.74^, 10^8.08^, and 10^8.37^ PFU/g, respectively (Supplementary Fig. 1b). These results indicate that the virus replication patterns differed between the 100-, 30-, and 10 μl-inoculation groups and the 3 μl-inoculation group. The virus in 3 ul of liquid may first replicate in the upper respiratory tract and then spread to the lower respiratory tract, whereas virus in at least 10 μl of liquid may directly reach the lower respiratory tract immediately after inoculation. Therefore, we decided to use 3 μl and 30 μl of virus liquid for the low- and high-volume inoculation systems, respectively, in this study.

**Distribution of inoculum in hamster respiratory tract.**

To determine the distribution of the inoculum in the hamsters, we inoculated hamsters intranasally with 30 μl or 3 μl of a tissue-marking dye. For both volumes, the tissue-staining dye was detected in the upper respiratory tract (Supplementary Fig. 2). In the 30-µl infection system, the tissue- marking dye was widely distributed in the nasal passage, whereas in the 3-µl infection system, the tissue-marking dye was distributed in only a limited area. In the 30-µl infection system, the dye was widely distributed in the esophagus, but in the 3-µl infection system, it was not detected in the esophagus. No dye was detected in the trachea or lungs. These results demonstrate that inoculation of 30 µl of liquid is sufficient to reach the esophagus and that microscopic droplets could have reached the lower respiratory tract.

**Multiple genes from the Omicron strain are involved in the limited replication of SARS-CoV-2 in the lower respiratory airways of hamsters.**

To identify other viral genes that may support the reduced replication in the lungs of hamsters infected with the Omicron variant, we generated a recombinant SARS-CoV-2 possessing the Omicron-E, -M or -N gene in the genetic background of the Wuhan strain [Wuhan-(E^Omicron^), Wuhan-(M^Omicron^), or Wuhan-(N^Omicron^)] (Supplementary Fig. 6a). No significant weight changes were observed in hamsters infected with any of these recombinant viruses (Supplementary Fig. 6a). We compared the titers of Wuhan-(E^Omicron^), Wuhan-(M^Omicron^), Wuhan-(N^Omicron^), Wuhan, and Omicron in hamster organs. On 3- and 5-days post-infection (dpi), all recombinant viruses were detected in the nasal turbinate and trachea (Supplementary Fig. 6b and 6c). While the Wuhan, Wuhan-(E^Omicron^), Wuhan-(M^Omicron^), and Wuhan-(N^Omicron^) viruses were recovered from the lungs of all infected animals on 3 dpi, Omicron was below the detection limit in the lungs of 2 of 4 infected hamsters on 3 dpi (Supplementary Fig. 6b). At 5 dpi, virus titers in the lungs of hamsters infected with Wuhan, Wuhan-(E^Omicron^), Wuhan-(M^Omicron^), or Wuhan-(N^Omicron^) were higher than those in hamsters infected with Omicron (Supplementary Fig. 6c). These results suggest that the E, M, or N gene derived from the Wuhan strain is not the single factor promoting the optimal spread of SARS-CoV-2 in the lower respiratory tract of hamsters.

Next, we generated a recombinant SARS-CoV-2 possessing the Omicron-ORF3, -ORF8, or -ORF10 gene in the genetic background of the Wuhan strain [Wuhan-(ORF3^Omicron^), Wuhan-(ORF8^Omicron^), or Wuhan-(ORF10^Omicron^)] (Supplementary Fig. 7a). No significant weight changes were observed in hamsters infected with any of these recombinant viruses (Supplementary Fig. 7a). We compared the titers of Wuhan-(ORF3^Omicron^), Wuhan-(ORF8^Omicron^), Wuhan-(ORF10^Omicron^), Wuhan, and Omicron in hamster organs. On 3 and 5 dpi, all recombinant viruses were detected in the nasal turbinate and traches (Supplementary Fig. 7b and 7c). While Wuhan, Wuhan-(ORF3^Omicron^), Wuhan-(ORF8^Omicron^), and Wuhan-(ORF10^Omicron^) were recovered from the lungs of all infected animals on 3 dpi, Omicron was below the detection limit in the lungs of all infected hamsters lung on 3 dpi (Supplementary Fig. 7b). At 5 dpi, virus titers in the lungs of hamsters infected with Wuhan, Wuhan-(ORF3^Omicron^), Wuhan-(ORF8^Omicron^), or Wuhan-(ORF10^Omicron^) were higher than those in hamsters infected Omicron (Supplementary Fig. 7c). These results suggest that the ORF3, ORF8, or ORF10 gene derived from the Wuhan strain is not the single factor promoting the optimal spread of SARS-CoV-2 in the lower respiratory tract of hamsters.

Together, our results suggest that several genes that are predicted to be adapted to humans are involved in the limited replication of SARS-CoV-2 in the lower respiratory tract of hamsters.

**Supplementary figure legends**

**Supplementary Fig. 1: Comparison of different infection volume systems in a hamster model using the Wuhan strain.**

Wild-type Syrian hamsters were intranasally inoculated with 10^3^ PFU of Wuhan strain virus in 100, 30, 10, or 3 μl. (**a**) Body weights of virus-infected (n=3) animals were monitored daily for up to 6 days. Data are presented as the mean percentages of the starting weight (±SD). (**b**) Virus titers in the organs of hamsters infected with each virus. Three hamsters per group were euthanized 3- and 6-days post-infection for virus titration. Virus titers in the lungs and nasal turbinate were determined by performing plaque assays in VeroE6/TMPRSS2 cells.

**Supplementary Fig. 2: Distribution of tissue-marking dye within the upper and lower respiratory tracts of hamsters.**

Tissue-marking dye was administered intranasally to two hamsters per group in a 30-μl or 3-μl volume. Thirty minutes later, the hamsters were euthanized, and gross examination of the respiratory tract was conducted. The examined tissues included the nasal passage, trachea (tr), esophagus (e), and lung. White arrows indicate tissue-marking dye observed in the nasal passage and esophagus.

**Supplementary Fig. 3: Body weights and Virus titers in the organs of hamsters infected with chimeric or parent viruses.**

**(a-c)**, Body weights of chimeric virus- and parent virus-infected (n=8) hamsters were monitored daily for 5 days. Note, three animals were euthanized in each group 3-days post-infection. Data are presented as the mean percentages of the starting weight (±SD). (**d-f)**, Virus titers in the organs of hamsters infected with each chimeric virus and the parent strains. For virus titration, four hamsters per group were euthanized 5-days post-infection. Virus titers in the nasal turbinate, trachea, and lung were determined by means of plaque assays in VeroE6/TMPRSS2 cells. Vertical bars show the average virus titers. Detection limits are indicated by dotted lines. Mean values were compared by an analysis of variance (ANOVA), followed by Tukey’s test (**p* <0.05, ***p* <0.01).

**Supplementary Fig. 4: A maximum likelihood (ML) tree of 575 SARS-CoV-2 genomes.**

Each branch color corresponds to its PANGO lineage shown in the right. The scale of the branch length is shown in the bottom. A red dot in an internal node indicates over 80% bootstrap support, while a blue dot indicates 50% to 80% bootstrap support.

**Supplementary Fig. 5: Omicron-unique amino acids in downstream of ORF3 substitutions are not found in other coronaviruses belonging to the subgenus Sarbecovirus.**

Among 60 representative coronaviruses belonging to the subgenus Sarbecovirus, we searched for viruses with E-T9I, M-Q19E, M-A63T or N-31-33del. A phylogenetic tree was constructed with RAxML-NG using the whole genome sequence with 100 bootstrap replicates. Red lines indicate the presence of Wuhan-type amino acids, blue lines indicate omicron-type amino acid substitutions, blank indicates omicron-type amino acid deletions, and the other colors indicate other amino acids.

**Supplementary Fig. 6: Body weights and virus titers in the organs of hamsters infected with recombinant Wuhan virus with its E, M or N gene derived from the Omicron strain.**

(**a**) Body weights of hamsters infected with chimeric or parent virus (each n=8), and monitored daily for 5 days. Note, four animals were euthanized in each group 3-days post-infection Data are presented as the mean percentages of the starting weight (±SD). (**b-c**) For virus titration, four hamsters per group were euthanized 3-days (**b**) and 5-days (**c**) post-infection. Virus titers in the nasal turbinate, trachea, and lung were determined by means of plaque assays in VeroE6/TMPRSS2 cells. Detection limits are indicated by dotted lines. Results obtained from each individual are shown in the same column. Mean values were compared by an analysis of variance (ANOVA), followed by Tukey’s test (**p* <0.05, ***p* <0.01).

**Supplementary Fig. 7: Body weights and virus titers in the organs of hamsters infected with recombinant Wuhan virus with its ORF3, ORF8, or ORF10 gene derived from the Omicron strain.**

(**a**) Body weights of hamsters infected with chimeric or parent virus (each n=8), and monitored daily for 5 days. Note, four animals were euthanized in each group 3-days post-infection Data are presented as the mean percentages of the starting weight (±SD). (**b**) For virus titration, four hamsters per group were euthanized 3-days (**b**) and 5-days (**c**) post-infection. Virus titers in the nasal turbinate, trachea, and lung were determined by means of plaque assays in VeroE6/TMPRSS2 cells. Detection limits are indicated by dotted lines. Results obtained from each individual are shown in the same column. Mean values were compared by an analysis of variance (ANOVA), followed by Tukey’s test (**p* <0.05, ***p* <0.01).
